# The Validation of the Parental Self-Efficacy Scale for Diabetes Management Among Parents of Children Wearing a Continuous Glucose Monitoring Sensor

**DOI:** 10.3390/biomedicines13061309

**Published:** 2025-05-27

**Authors:** Áron Hölgyesi, Andrea Luczay, Péter Tóth-Heyn, Eszter Muzslay, Eszter Világos, Attila J. Szabó, Petra Baji, Levente Kovács, László Gulácsi, Zsombor Zrubka, Márta Péntek

**Affiliations:** 1 Health Economics Research Center, University Research and Innovation Center, Obuda University, 1034 Budapest, Hungarypentek.marta@uni-obuda.hu (M.P.); 2 Pediatric Center, Semmelweis University, 1083 Budapest, Hungary; 3 Musculoskeletal Research Unit, Bristol Medical School, University of Bristol, Bristol BS10 5NB, UK; 4 Physiological Controls Research Center, University Research and Innovation Center, Obuda University, 1034 Budapest, Hungary; 5 Doctoral School of Innovation Management, Obuda University, 1084 Budapest, Hungary

**Keywords:** pediatric diabetes, glucose control, parental self-efficacy, patient-reported outcome measures, capability well-being, health-related quality of life

## Abstract

**Background/Objectives:** Parental involvement is essential in managing type 1 diabetes mellitus (T1DM) in children, particularly with the growing use of continuous glucose monitoring (CGM). Validated tools assessing parental self-efficacy in this context remain limited. This study aimed to validate the Parental Self-Efficacy Scale for Diabetes Management (PSESDM) among parents of children using a CGM sensor and to examine its associations with diabetes outcomes and parental characteristics. **Methods:** A cross-sectional study was conducted involving 106 parent–child dyads at a university pediatric diabetes center. Parents completed the Hungarian PSESDM. Data regarding children’s HbA1c level were recorded, along with standard measures of their general and diabetes-specific quality of life (EQ-5D-Y-3L, PedsQL Diab); data regarding parents’ health literacy (Chew), fear of hypoglycemia (HFS), health-related quality of life (EQ-5D-5L), and capability well-being (ICECAP-A) were also collected. The PSESDM’s reliability, internal consistency, and discriminant and criterion validity were assessed using standard statistical methods. **Results:** The PSESDM demonstrated good internal consistency (Cronbach’s α = 0.857) and strong item–total correlations (range: 0.678–0.791). Higher parental self-efficacy was significantly associated with better glucose control (lower HbA1c, r_s_ = −0.50) and weakly correlated with the child’s diabetes-specific quality of life (r_s_ = 0.20). Among parental characteristics, self-efficacy correlated strongly with capability well-being (r_s_ = 0.52), moderately with health literacy (r_s_ = −0.30), and showed no difference between socio-demographic subgroups, except for the subgroup related to income. **Conclusions:** The PSESDM is a valid and reliable tool for measuring self-efficacy in parents of children with T1DM using CGM sensors. Its associations with children’s HbA1c levels, diabetes-specific quality of life, and parental characteristics support its clinical relevance and potential use in identifying families at risk for poorer diabetes outcomes.

## 1. Introduction

The management of type 1 diabetes mellitus (T1DM) is a highly complex task. In addition to its appropriate pharmacological treatment, numerous other factors require daily attention, such as maintaining a suitable diet, regular blood glucose monitoring, consistent physical activity, and adjusting insulin dosage according to individual needs. This complexity places a particularly heavy burden on patients and caregivers, especially in the case of pediatric type 1 diabetes, where children require increased parental supervision and support to manage their lifestyle and daily tasks associated with their disease [1]. Moreover, advanced diabetes therapies, such as insulin pumps and continuous glucose monitoring (CGM) sensors, are becoming increasingly used, offering numerous short- and long-term benefits in terms of diabetes outcomes and the maintenance of patients’ balanced health state [2]. These advantages include a reduction in the frequency of hypo- and hyperglycemic episodes, better control of nighttime blood glucose levels, a decreased risk of cardiovascular complications, and improved health-related quality of life (HRQoL) [3,4,5,6]. However, the effective use of these devices demands proper knowledge, preparedness, and frequent intervention, all of which further enhances the significance of the parents’ role [7,8]. Therefore, to achieve the desired outcomes, both children and their parents need to exhibit the appropriate competencies and confidence in their abilities to sufficiently manage the disease and related tasks, often referred to as self-efficacy.

According to its general definition, self-efficacy is a person’s belief in his or her own ability to perform specific tasks and behaviors, with the aim of achieving the desired outcomes. Since its initial description by Bandura et al., the concept has been extended to healthcare as well, and the importance of self-efficacy has been investigated in the management of numerous health conditions, including diabetes [9]. Among adults and adolescents, it has been clearly established that higher self-efficacy leads to better adherence and improved glycemic control [10,11]. Despite the growing attention to the role of caregivers and parents in managing childhood diabetes in recent years, parental self-efficacy has remained relatively under-researched. The limited number of studies conducted so far suggest that children of parents with higher self-efficacy are better able to manage their condition and tend to have better glycemic control and diabetes-related quality of life [12].

Despite its obvious importance, validated tools specifically designed to measure parental self-efficacy in the context of childhood type 1 diabetes are scarce. Available instruments differ in several fundamental characteristics that may influence their practical applicability, e.g., the number of items included or the specific parental subgroups they target [12,13,14]. Recently, the Parental Self-Efficacy Scale for Diabetes Management (PSESDM), originally developed in English, was introduced as a general tool applicable to parents of children with T1DM. It assesses parental self-efficacy through a total of eight items, allowing for quick and easy administration. To date, its psychometric properties have only been evaluated in a single, small-sample study, which confirmed the scale’s validity and provided basic information about its reliability, but did not assess its association with parental characteristics beyond a limited set of demographic variables [15].

Since its initial publication, the scale has been increasingly used in studies examining the association between children’s T1DM outcomes and parental self-efficacy and health literacy [16,17], the effects of social interventions on how parents cope with their child’s diabetes [18], and the mediating role of spirituality in parents of children newly diagnosed with T1DM [19]. Furthermore, it has been employed as a standard reference measure in studies evaluating caregiver adjustment, parental benefit finding, and diabetes-specific routines [20,21,22]. Despite the sound methodology of these studies, they were relatively heterogeneous in terms of data reporting quality. Basic information on the PSESDM was often missing, including data regarding its association with demographic correlates, HbA1c levels, and—most notably—the use of advanced digital health technologies such as insulin pumps and CGM sensors. Consequently, current knowledge about the psychometric performance of the scale remains limited, especially in subgroups utilizing state-of-the-art treatment options. Additionally, the PSESDM has not yet been evaluated in relation to other parental characteristics that have previously been shown to correlate with self-efficacy and expected outcomes, such as the parent’s HRQoL and well-being [23,24,25]. In addition to their clinical relevance, the assessment of these factors is also gaining importance in economic evaluations, particularly when determining the health and social benefits associated with advanced digital treatment options and interventions [26,27]. The EQ-5D-5L has long been a standard tool for this purpose; however, the ICECAP-A questionnaire has received growing attention and is increasingly being applied in health economic analyses [28]. It was designed to assess capability well-being, which captures a broader range of life aspects—such as autonomy, achievement, and social participation—making it particularly relevant for evaluating the broader impact of interventions beyond the health-related aspects alone. Nevertheless, despite their clear relevance, data on these measures are often unavailable or incomplete [29]. In such cases, it becomes necessary to estimate missing values based on the information already available [30]. Therefore, studies providing insights into the applicability of the EQ-5D-5L and the ICECAP-A in the context of pediatric T1DM, along with their relationships with parental self-efficacy and key outcomes, are needed.

The primary aim of this research was to validate the PSESDM and examine its psychometric properties among parents of children using CGM sensors. Additionally, this study sought to expand on previous works by investigating a broad range of socio-demographic variables and other parental characteristics, including health literacy, fear of hypoglycemia, HRQoL, and well-being.

## 2. Methods

### 2.1. Data Collection

A cross-sectional survey was carried out in a single university clinical diabetes center with the aim of assessing the quality of life and attitudes of children suffering from type 1 diabetes mellitus and their caregiving parents. Results regarding parental electronic health literacy and its impact on diabetes management and outcomes have been published separately [17]. In brief, parent–child pairs were recruited by treating diabetologists. Inclusion criteria required that the parent be at least 18 years old and reside with the child at least part time and that the child had been diagnosed with type 1 diabetes mellitus for at least three months.

Upon study entry, the participants were informed that involvement was voluntary, their data would remain non-personal and anonymous, and that the data would only be used for the scientific purposes defined in the study protocol. Written informed consent was obtained from all participants at the time of involvement. Ethical approval was obtained from the Hungarian Medical Research Council (IV/3848-1/2021/EKU; BMEÜ/1620-1/2022/EKU).

The survey comprised three modules, which were administered separately. In the first module, information was collected from parents regarding their socio-demographic background (sex, age, education, type of residence, family status, employment, net per capita income) and their child (sex, age, childcare circumstances). Also, the Hungarian version of standard measurement tools were used alongside the PSESDM, evaluating general health literacy (Chew questionnaire), fear of hypoglycemia (Hypoglycemia Fear Survey), general HRQoL (EQ-5D-5L), and capability well-being (ICECAP-A).

The second module was administered to the children and obtained information concerning their general HRQoL (EQ-5D-Y-3L) and diabetes-specific quality of life (PedsQL Diab 3.0).

The third module involved treating diabetologists, who provided medical information about the child’s disease characteristics, including disease duration, duration of care at the clinical center, type of insulin treatment (pen or pump), CGM usage, current HbA1c level (recorded as a percentage), diabetes-related acute events or device malfunction, chronic complications of diabetes, and comorbidities.

### 2.2. Standard Patient-Reported Outcome Measures (PROMs) Used in the Study

#### 2.2.1. Parental Self-Efficacy Scale for Diabetes Management (PSESDM)

The PSESDM assesses how effective parents perceive themselves to be in managing their child’s type 1 diabetes [15]. It was originally adapted from the Perceived Diabetes Self-Management Scale (PDSMS), which was modified by replacing “my diabetes” with “my child’s diabetes”, and by slightly simplifying its wording [31]. The PSESDM consists of eight statements regarding the parent’s perceived effectiveness in performing diabetes-related tasks. Parents are asked to indicate their level of agreement with each statement using a five-point Likert scale ranging from 1 (strongly disagree) to 5 (strongly agree). Four out of the eight statements are negatively worded, meaning that stronger agreement indicates lower self-efficacy. For these items, reverse scoring is applied. The total score is calculated by summing the numeric values of all responses, resulting in a score that ranges from 8 to 40. Higher scores indicate greater parental self-efficacy.

In this study, we used the Hungarian-language version of the PSESDM, developed by our research team. During the forward translation process, multiple independent translations were produced by the researchers involved. These versions were then reviewed and reconciled to resolve discrepancies and to produce a consolidated version. This version was back-translated into English by two independent experts, and the back-translations were compared with the original English version by the research team to ensure accuracy and consistency. The Hungarian version draft was piloted as part of the full questionnaire survey involving clinical experts and parents. Following proofreading and a final quality check, the finalized version was approved for use.

#### 2.2.2. Chew Questionnaire

The Chew questionnaire was developed to identify people with limited health literacy in the clinical setting [32,33]. It consists of three questions regarding how confident respondents feel that they can (1) fill in forms independently, (2) whether they need help with the interpretation of hospital documents, and (3) whether they have problems with understanding them. Responses can be provided using a five-point ordinal scale, ranging from 0 (never) to 4 (always). The scores given to each question are summed up to calculate the total score, ranging from 0 to 12, with higher scores indicating worse health literacy.

#### 2.2.3. Hypoglycemia Fear Survey

The Hypoglycemia Fear Survey (HFS) is a reliable tool for measuring parents’ concerns and behaviors related to hypoglycemic episodes in their children with type 1 diabetes mellitus [34]. It consists of two subscales: the first focuses on parental behaviors aimed at avoiding hypoglycemic episodes (10 statements), while the second assesses worries about hypoglycemic episodes and their possible consequences (15 statements). Respondents indicate how true each statement feels for them on a five-point scale (0—never; 4—almost always). To calculate the final score (ranging from 0 to 100), the individual scores for each statement are summed. A higher score indicates a greater fear of hypoglycemia. Based on the data from this study, the Hungarian version of the HFS showed a good internal consistency (Cronbach’s alpha = 0.859).

#### 2.2.4. EQ-5D-5L

The EQ-ED-5L is a measure of general HRQoL [35]. It assesses the health status of respondents in five domains: mobility, self-care, usual activities, pain/discomfort, and anxiety/depression. The severity of current problems in each domain are rated on a five-point scale, ranging from 1 (no problem) to 5 (unable to). The reported problem levels are combined with preference weights to calculate the EQ-5D-5L index, which represents the utility of a given health state from a societal perspective. In the present study, the Hungarian national tariffs were used to calculate the index values (range: −0.848–1.000) [36]. In addition to the descriptive system, the EQ-5D-5L includes a visual analogue scale (EQ VAS), on which respondents can indicate their current health status. It ranges from 0 to 100, where 0 represents the worst and 100 the best health that the respondent can imagine.

#### 2.2.5. ICECAP-A

The ICECAP-A questionnaire was designed to assess the capability well-being of individuals aged 18 years and over [37]. It covers five domains (attachment, stability, achievement, enjoyment, and autonomy), with one question representing each domain. Respondents can rate their current well-being using a four-level Likert scale, where 1 indicates no capability, and 4 indicates full capability. The ICECAP-A index score is calculated by combining the reported capability levels with corresponding preference weights. For this study, the Hungarian value set was applied to derive the index scores [38].

#### 2.2.6. EQ-5D-Y-3L

The EQ-5D-Y-3L measures the general health-related quality of life of children [39]. It comprises five questions covering five domains of health (mobility; taking care of myself; doing usual activities; feeling pain or discomfort; feeling worried, sad, or unhappy). The level of current health problems in each domain is indicated on a three-level scale: 1—no problems; 2—some problems; 3—a lot of problems. Index values are obtained using national value sets. In this study, the Hungarian tariffs were used for the calculation [40]. EQ-5D-Y-3L also includes a visual analogue scale (EQ VAS), ranging from 0 (worst imaginable health) to 100 (best imaginable health), through which respondents can indicate their current health status.

#### 2.2.7. PedsQL Diab

The PedsQL questionnaire’s Diabetes Module (PedsQL Diab) was designed to assess diabetes-specific quality of life in children [41]. It consists of 28 questions divided across five domains: physical functioning (8 questions), emotional functioning (5 questions), social functioning (5 questions), and school functioning (5 questions). Each question describes a specific problem related to diabetes, and respondents are asked to indicate how often they have experienced that particular issue in the last month. Responses are recorded on a five-level ordinal scale ranging from 0 (never) to 4 (almost always). To calculate the final score, responses are reverse-scored and transformed to a 0–100 scale (0 = 100; 1 = 75; 2 = 50; 3 = 25; 4 = 0), and the average of all item scores is taken. Higher scores reflect better diabetes-specific quality of life. This study used version 3.0 of the questionnaire [42].

### 2.3. Statistical Analysis

The sample characteristics and the distribution of the PSESDM scores and item-level responses were analyzed using descriptive methods. Summary statistics of other standard PROMs used in the study were also calculated for the total sample and by socio-demographic subgroups and disease characteristics. The glycemic target for the HbA1c level was approved as <7.0%, according to the recommendation of the International Society for Pediatric and Adolescent Diabetes (ISPAD) [43].

The internal consistency and reliability of the PSESDM scale was examined by assessing how the individual items relate to each other (inter-item Spearman’s correlation) and to the scale’s total score (item-total score Spearman’s correlations). To eliminate bias and provide a more accurate evaluation of each item’s contribution to the overall scale, corrected item–total score Spearman’s correlations were also calculated, which reflect each item’s contribution to the total score, after excluding the item itself. In addition, the Cronbach’s alpha was computed to provide an overall estimate of the scale’s internal consistency (excellent: α ≥ 0.9; good: 0.9 > α ≥ 0.8; acceptable: 0.8 > α ≥ 0.7) [44].

The association between PSESDM scores and socio-demographic variables was examined to assess the scale’s discriminant validity. Differences by subgroups were compared using the Mann–Whitney-U and Kruskal–Wallis tests for binary and multiple groups, respectively.

Concurrent validity, a type of criterion-related validity, refers to the extent to which a measurement is associated with other measures of traits or outcomes that are theoretically related and assessed at the same point in time [45]. To assess concurrent validity, the association of the PSESDM with other standard PROMs measuring parental characteristics (Chew questionnaire, HFS, EQ-5D-5L, ICECAP-A), children’s general HRQoL (EQ-5D-5L), and diabetes outcomes (PedsQL Diab, HbA1c level) was evaluated by calculating Sperman’s correlations between the scales’ total scores (strong: r_s_ ≥ 0.5; moderate: 0.5 > r_s_ ≥ 0.3; weak: r_s_ < 0.3) [46]. To gain a deeper insight into the observed associations, an item-level analysis was conducted by assessing the dispersion of standard PROM scores and HbA1c values across response levels for each item of the PSESDM.

The statistical significance was approved as *p* < 0.05 in all cases. The full analysis was performed in Stata version 17.0 (StataCorp LCC, College Station, TX, USA).

## 3. Results

### 3.1. Sample Characteristics

Detailed results of the socio-demographic and disease characteristics of the sample are shown in Table 1. All in all, 106 parent–child pairs using a CGM sensor were involved in the study. The majority of parents were women (79.3%), belonged to the age category 40–49 (73.6%; mean age 42.9 ± 5.0 years), had a tertiary education (51.9%), lived in an urban environment (50.9%), were married (71.7%), and were fully employed (67.0%). More than two-thirds had a monthly net per capita income less than or equal to EUR 1000. Parents’ mean scores on the Chew questionnaire, the Hypoglycemia Fear Survey (HFS), the EQ-5D-5L, and the ICECAP-A questionnaires were 2.3 ± 1.8, 41.4 ± 12.9, 0.97 ± 0.07, and 0.89 ± 0.12, respectively, with no meaningful difference between the examined subgroups.

The children were between 11 and 14 years of age (71.6%; mean age 11.7 ± 1.8 years). They had been suffering from T1DM for a mean of 5.2 ± 2.7 years (range: 1–13) and had been treated at this clinic for a mean of 4.6 ± 2.4 years (range: 1–13). The share of insulin pump and pen users was nearly equal (48.1% and 51.9%, respectively), with no meaningful differences between the two groups in terms of demographic and disease characteristics. At the time of the study, the mean HbA1c was 7.3 ± 1.0% (range: 5.4–10.0) in the sample, and nearly two-thirds of children (61.3%) were above the ISPAD-defined HbA1c target (<7.0%). The presence of a comorbidity was reported in N = 33 cases, with N = 2 children having two comorbidities simultaneously. The reported conditions were coeliac disease (N = 12), thyroid disorder (N = 20), juvenile idiopathic arthritis (N = 1), congenital adrenal hyperplasia (N = 1), and epilepsy (N = 1). One case of adverse event (device malfunction) and chronic diabetes complication (diabetic nephropathy) were recorded, respectively. Children’s mean scores on the EQ-5D-Y-3L and the PedsQL Diab questionnaires were 0.95 ± 0.09 and 74.7 ± 11.5, respectively, which did not differ between any of the examined subgroups.

### 3.2. Self-Efficacy by PSESDM Items

**Figure 1:**
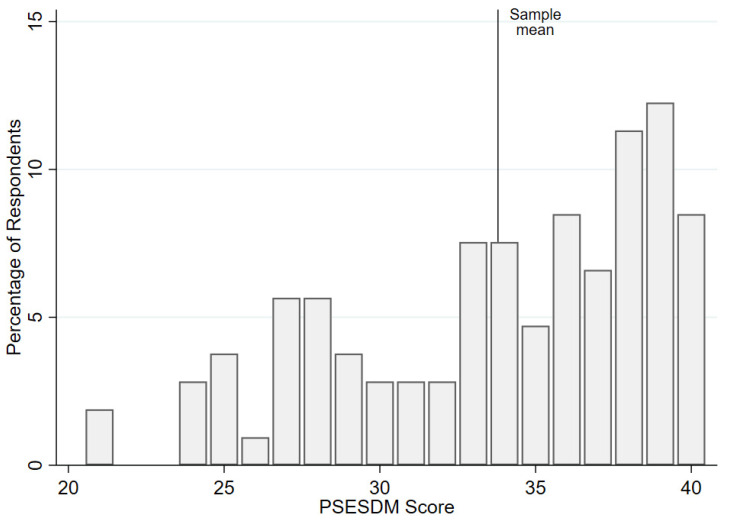
Dispersion of the PSESDM scores in the study sample.

As shown in Figure 1, the distribution of PSESDM scores in the total sample was left-skewed, with a mean score of 33.8 ± 5.0 (range: 21–40).

**Figure 2:**
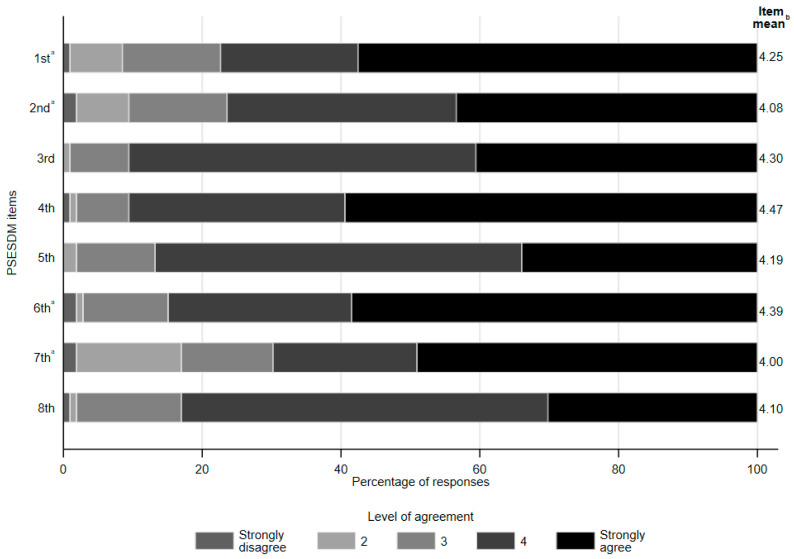
Share of responses and mean scores by the PSESDM items. ^a^ Items are reversely scored. ^b^ Higher mean score indicates greater perceived self-efficacy for the respective item The eight items of the PSESDM [15] are as follows: 1. It is hard for me to find ways to solve problems that occur in dealing with my child’s diabetes. 2. When I try to change things I don’t like about my child’s diabetes, it doesn’t work. 3. I take care of my child well when it comes to his/her diabetes. 4. I am able to deal with things related to my child’s diabetes as well as others. 5. I am successful when it comes to projects I do to take care of my child’s diabetes. 6. Usually, my plans to take care of my child’s diabetes don’t work out. 7. No matter how hard I try, taking care of my child’s diabetes doesn’t turn out the way I like. 8. I’m usually able to accomplish the goals I set in trying to take care of my child’s diabetes.

Regarding responses to individual PSESDM items, parents were shown to be the most efficacious in dealing with things related to their child’s diabetes, as well as others (4th item—mean score 4.47 ± 0.76), followed by accomplishing plans to take care of their child’s diabetes (6th item—mean score 4.39 ± 0.88) and taking care of their child well when it comes to his/her diabetes (3rd item—mean score 4.30 ± 0.66). In contrast, the lowest efficacy was observed in taking care of the child’s diabetes according to the parent’s preferences (7th item—mean score 4.00 ± 1.19), followed by trying to change things they do not like about their child’s diabetes (2nd item—mean score 4.08 ± 1.02) and accomplishing the goals they set in trying to take care of their child’s disease (8th item—mean score 4.1 ± 0.76). No meaningful difference in the distribution of responses was observed between parental socio-demographic subgroups. Detailed results are shown in Figure 2.

### 3.3. Internal Consistency and Reliability

The PSESDM items correlated significantly with each other with a mean of r_s_ = 0.48 (range: 0.359–0.599), indicating a moderate inter-item relationship within the scale (Table S1). Item–total score correlations (range: 0.678–0.791) and corrected item–total score correlations (range: 0.590–0.671) were strong, suggesting good reliability. The Cronbach’s alpha was 0.857 for the full scale, and no further improvement was observed when deleting any of the items, indicating good internal consistency. Detailed results are shown in Table 2.

### 3.4. Associations with Socio-Demographic Characteristics, Treatment Modality, and Health Status

The dispersion of the PSESDM scores by socio-demographic subgroups and disease characteristics are shown in Table 1. The analysis revealed no significant difference in the PSESDM scores, except for per capita income, where higher PSESDM scores were observed in the higher income categories. A slight increasing trend in PSESDM scores was seen by educational level, but the difference was not statistically significant. Regarding the children’s characteristics, the PSESDM scores did not significantly differ by their age or gender. Higher PSESDM scores were observed among parents whose child’s HbA1c value was within the therapeutic target range (less than 7.0%) (N = 41; mean score 36.3 ± 3.8) compared to those whose child’s HbA1c was outside this range (N = 65; mean score 32.2 ± 5.1). No difference was observed for the type of insulin treatment or for other indicators of the child’s health status, such as the presence of a comorbidity or adverse event.

### 3.5. Associations with Parental Characteristics, Children’s Quality of Life, and Diabetes Outcomes

**Figure 3:**
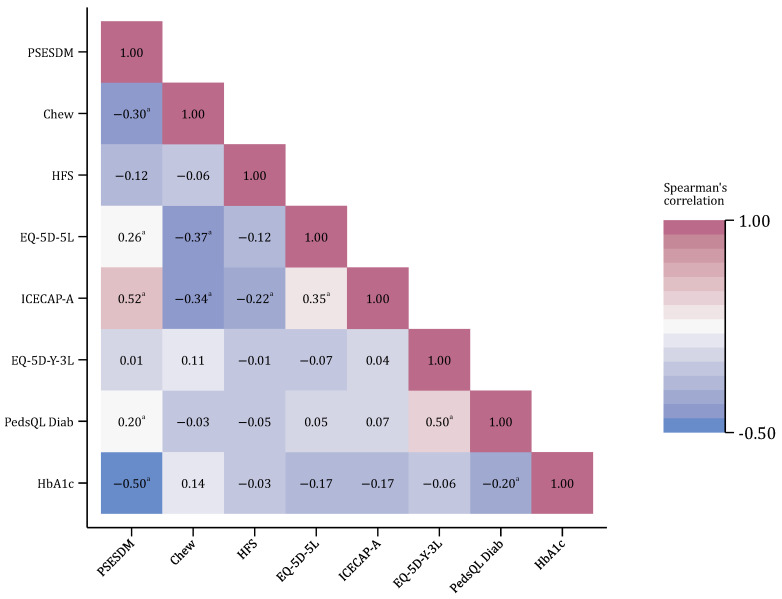
Correlations between the PSESDM and other standard measures. The number of observations was N = 106 in all cases; ^a^ *p* < 0.05.

The PSESDM total score correlated moderately with the Chew questionnaire (health literacy) and weakly with the EQ-5D-5L (general health-related quality of life), while its association with the ICECAP-A (capability well-being) was strong. The correlation with the HFS measure (fear of hypoglycemia) was not significant. Regarding the child’s health status and disease outcomes, no association was found between the PSESDM and the EQ-5D-Y-3L (general health-related quality of life), while its correlation was weak with the PedsQL Diab score (diabetes-specific quality of life) and strong with the child’s HbA1c level. Results of the correlation analysis are shown in Figure 3.

The item-level analysis of the standard questionnaire’s score distribution revealed that the ICECAP-A scores were significantly associated with all the PSESDM items, while the Chew questionnaire was associated with four items (Item 1, Item 2, Item 4, and Item 5) and the EQ-5D-5L with three items (Items 1–3). The child’s HbA1c level was also associated with all the PSESDM items, except for Item 6. In contrast, the PedsQL Diab scores were not associated with the reported level of self-efficacy in any of the PSESDM items. Detailed results are shown in Table S2.

## 4. Discussion

The present study successfully validated the Parental Self-Efficacy Scale for Diabetes Management (PSESDM) and examined its reliability and internal consistency among parents of children using continuous glucose monitoring (CGM) sensors. To the best of our knowledge, this is the first study to evaluate the PSESDM in this specific subgroup. Alongside the assessment of a broad range of socio-demographic variables, we also evaluated the scale’s psychometric properties in relation to children’s health status and key clinical outcomes, including HbA1c levels and diabetes-related quality of life. Furthermore, we are the first to investigate the association between the PSESDM and several standardized measures of parental characteristics, such as the preference based EQ-5D-5L and the ICECAP-A questionnaires. Our findings indicate that higher parental self-efficacy is associated not only with better child health outcomes but also with improved parental well-being.

Our study provides important descriptive data regarding how parents of children wearing a CGM sensor perceive their self-efficacy across different domains covered by the PSESDM. The highest level of confidence was reported in handling diabetes-related tasks as effectively as other duties. Conversely, parents felt the least confident in managing their child’s diabetes according to their own preferences. While average item scores showed only minor differences (range: 4.00–4.47) and no formal statistical comparison was conducted, these findings offer an insight into the areas where parents may struggle the most. The results have important implications for healthcare professionals, as they can help them identify specific domains in which additional support or education may be needed.

A key strength of the present study is the more in-depth analysis of the PSESDM’s internal consistency and reliability compared to that observed in previous research. The Cronbach’s alpha was 0.857, slightly higher than the 0.84 reported by Marchante et al., confirming good internal consistency [15]. Additionally, to provide a more detailed understanding of the scale’s structure, we examined item–item and item–total correlations. These were consistently strong, with minimal variation across items, suggesting a balanced structure in which all items contribute meaningfully and fairly equally to the scale’s total score.

Our observations regarding the association between socio-demographic variables and PSESDM scores differ from previously published results in that we found no meaningful differences between socio-demographic subgroups. In contrast, earlier studies reported a positive association between parental education level and PSESDM scores [17]. A possible explanation for this deviation is that CGM use itself is more common among the more highly educated [17]. Since our study focused exclusively on CGM users, the effect of education level may have been attenuated. Accordingly, the proportion of parents with tertiary education in the sample was relatively high compared to that in previous studies [17]. It is also notable that no difference was found in the PSESDM scores regarding children’s demographic characteristics or type of insulin treatment (pen or pump). To our knowledge, this latter association has not been previously examined. This is a particularly important finding because insulin pumps are advanced devices that allow for improved control and convenience. However, this advantage does not appear to translate into increased parental self-efficacy. The underlying reasons for this phenomenon may deserve further investigation in future targeted studies.

The criterion validity of the PSESDM has been established in relation to diabetes outcomes such as HbA1c and diabetes-specific quality of life [15,21]. Our results confirmed these findings, as parents of children with HbA1c levels under the ISPAD-defined target (>7.0%) showed significantly higher self-efficacy compared to those above this threshold [43]. This was further supported by the correlation analyses, in which higher PSESDM scores were associated with lower HbA1c values, indicating better glycemic control. No significant differences in self-efficacy were observed with respect to diabetes-related complications or comorbidities, which was somewhat expected due to their relatively low occurrence in the sample. While a correlation was observed between the PSESDM total score and diabetes-specific quality of life, as measured by the PedsQL Diabetes module, this relationship did not persist in the more detailed item-level analysis, which can be explained by the fact that the correlation between the two instruments’ total scores was rather weak.

Another strength of our study is that it extended the assessment of the PSESDM’s criterion validity beyond children’s clinical outcomes to include key parental characteristics. The strongest correlation was found with capability well-being, as measured by the ICECAP-A, suggesting that parents with greater overall well-being and life satisfaction also feel more competent in managing their child’s diabetes. This is a notable contribution to the literature, as the association between parental well-being and self-efficacy in pediatric diabetes has been largely unexplored, and to our knowledge, this is the first study to examine the ICECAP-A in this context [47]. We also assessed the relationship between parental HRQoL and self-efficacy, as previous studies described that—along with several other factors—higher self-efficacy is a significant predictor of better HRQoL among parents of children with T1DM. Our findings appear to support this relationship, as we observed a weak positive correlation between the EQ-5D-5L and the PSESDM. We must highlight that we were not only the first to evaluate the measurement properties of PSESDM in relation to parental HRQoL, but also the first to use the EQ-5D-5L for this purpose. Therefore, our study provides the first evidence regarding the association between this widely used standard measure of general HRQoL and parental self-efficacy in the context of pediatric T1DM [23]. An important practical implication of the present study is that exploring the relationships between the PSESDM and the ICECAP-A and EQ-5D-5L may support health economic analyses in cases where no data are available from these preference-based measures, and the estimation of missing values is necessary. The results obtained from our sample of 106 parents are promising, and further exploration in larger samples would be recommended. This is particularly relevant for the future, as the evaluation of digital health interventions is receiving increasing attention in health technology assessment, and in most European countries, the submission of PROM data is required to demonstrate clinical effectiveness [48]. Additionally, although beyond the scope of the present study, it may be worthwhile in the future to determine the causal relationship between parental characteristics. That is, whether inherently higher HRQoL and well-being makes more effective caregivers, or conversely, whether effective diabetes management (and its impact on the child’s condition) leads to an improvement in the parents’ condition.

Another important observation is the significant association between parental self-efficacy and general health literacy, as a negative correlation (r_s_ = −0.30) was found between the PSESDM and the Chew questionnaire scores (where lower Chew scores indicate better health literacy). This finding highlights that the improvement in parental awareness and knowledge may lead to the increased efficacy of care and consequently, better diabetes outcomes. It also underscores the broader importance of effective communication between clinicians and caregivers, as well as the need for decision makers to support programs aimed at improving patient and caregiver education.

Some limitations of our study should to be noted. Recruitment was conducted in one hospital-based center, which might cause selection bias in the sample and limit the generalizability of the results. We did not formally evaluate construct validity. However, results have previously been reported which showed that the scale is appropriate in this regard. As we obtained good results for internal consistency, we assume that the construct validity of the scale would not differ significantly from that reported in previous studies. Nevertheless, we acknowledge that an in-depth analysis of the construct validity of the Hungarian PSESDM would be a valuable avenue for future comprehensive validation. There are additional measurement properties that should be addressed in future research, such as the minimal important difference (MID) and the scale’s responsiveness and ability to detect change over time. Although our study design did not allow for the evaluation of these properties, they are essential for the practical application of the PSESDM in clinical settings, particularly for monitoring change and identifying parents in need of additional support.

## 5. Conclusions

In conclusion, our study confirmed that the PSESDM is a valid and appropriate tool for assessing self-efficacy among parents of children with T1DM wearing a CGM sensor. The results demonstrate that the PSESDM score is significantly associated with clinically relevant child diabetes outcomes and parental characteristics, including capability well-being. Our findings underscore the clinical applicability of the PSESDM and suggest that it may be a useful tool in the future for identifying patient and parent groups who are at greater risk of worse outcomes, thereby guiding clinical attention and intervention. These results contribute to evidence-based healthcare decision making and may serve as input for designing public health and social interventions.

## Figures and Tables

**Table 1:** Socio-demographic and disease characteristics of the sample.

	N	%	PSESDM Score		
			Mean (SD)	Median (Range)	
Total	106	100	33.8 (5.0)	35 (21–40)	-
Parents					
Sex					
Female	84	79.3	33.6 (5.2)	34.5 (21–40)	*p* = 0.667
Male	22	20.7	34.5 (4.4)	35.5 (25–40)	Χ^2^(1) = 0.185
Age					
30–39	24	22.6	32.5 (5.9)	32.5 (21–40)	*p* = 0.415
40–49	78	73.6	34.2 (4.8)	35.5 (21–40)	Χ^2^(3) = 2.852
50–59	3	2.8	34.7 (2.3)	36 (32–36)	
60–69	1	0.9	27.0 (-)	27(-)	
Education					
Primary	4	3.8	31.3 (6.5)	30 (25–40)	*p* = 0.165
Secondary	47	44.3	32.9 (5.4)	33 (21–40)	Χ^2^(2) = 3.603
Tertiary	55	51.9	34.8 (4.5)	36 (21–40)	
Residency					
Capital	31	29.3	34.7 (5.0)	36 (21–40)	*p* = 0.429
Urban	54	50.9	33.6 (4.6)	34 (24–40)	Χ^2^(2) = 1.694
Rural	21	19.8	33.0 (6.2)	35 (21–40)	
Family status					
Married	76	71.7	34.0 (5.0)	35.5 (21–40)	*p* = 0.478
Domestic relationship	14	13.2	34.9 (4.3)	36.5 (25–40)	Χ^2^(4) = 3.502
Single	5	4.7	31.0 (7.8)	27 (24–40)	
Divorced	8	7.6	31.3 (4.5)	31 (26–39)	
Other	3	2.8	34.0 (5.0)	34 (29–39)	
Employment					
Full-time	71	67.0	34.6 (4.6)	36 (21–40)	*p* = 0.098
Part-time	18	17.0	31.2 (5.4)	29 (24–40)	Χ^2^(5) = 9.279
Retired	2	1.9	30.0 (4.2)	30 (27–33)	
Unemployed	2	1.9	30.0 (1.4)	30 (29–31)	
Homemaker	7	6.6	32.6 (7.3)	33 (21–40)	
Other	6	5.7	36.0 (5.0)	37 (27–40)	
Net per capita income (EUR)					
<500	35	33.0	31.7 (5.5)	32 (21–40)	*p* = 0.003
501–1000	37	34.9	34.0 (4.0)	35 (25–40)	Χ^2^(3) = 13.742
1001–1500	11	10.4	37.4 (3.7)	39 (27–40)	
>1500	1	0.9	27.0 (-)	27 (-)	
Missing	22	20.8	-	-	
Children					
Sex					
Female	52	49.1	34.8 (4.4)	36 (25–40)	*p* = 0.072
Male	54	50.9	32.8 (5.5)	33 (21–40)	Χ^2^(1) = 3.230
Age					
7–10	27	25.5	33.6 (5.3)	35 (24–40)	*p* = 0.965
11–14	76	71.7	33.9 (5.0)	35 (21–40)	Χ^2^(2) = 0.072
15–18	3	2.8	34.0 (6.0)	34 (28–40)	
Treatment					
Pen + CGM	55	51.9	32.9 (5.2)	34 (21–40)	*p* = 0.100
Pump + CGM	51	48.1	34.7 (4.7)	36 (21–40)	Χ^2^(1) = 2.709
HbA1c below target (>7.0%)					
Yes	41	38.7	36.3 (3.8)	37 (24–40)	<0.001
No	65	61.3	32.2 (5.1)	33 (21–40)	Χ^2^(1) = 1.905

Percentages may not add up to 100.0% due to rounding. *p*-values, Chi-squared statistics, and degrees of freedom (in parentheses) are reported for binary and multiple group comparisons carried out using the Mann–Whitney U and Kruskal–Wallis tests, respectively.

**Table 2:** Reliability and internal consistency of the PSESDM questionnaire.

	Item–Total Score Correlation	Corrected Item–Total Score Correlation ^a^	Cronbach’s Alpha, If Item Is Deleted
Item 1	0.733	0.645	0.842
Item 2	0.741	0.647	0.830
Item 3	0.678	0.590	0.846
Item 4	0.692	0.629	0.840
Item 5	0.743	0.660	0.837
Item 6	0.694	0.596	0.842
Item 7	0.791	0.671	0.832
Item 8	0.734	0.622	0.847
Full scale	-	-	0.857

^a^ Correlation with the scale’s total score if the item is deleted; coefficients represent Spearman’s correlation.

## Data Availability

The original contributions presented in this study are included in the article. Further inquiries can be directed to the corresponding author.

## Supplementary Materials

The following supporting information can be downloaded at: https://www.mdpi.com/article/10.3390/biomedicines13061309/s1, Table S1: Inter-item correlations (Spearman’s Rho); Table S2: The item-level distribution of standard questionnaires and HbA1c values.

## Abbreviations

The following abbreviations are used in this manuscript:

CGM	continuous glucose monitoring
HRQoL	health-related quality of life
PROM	Patient-Reported Outcome Measure
PSESDM	Parental Self-Efficacy Scale for Diabetes Management
T1DM	type 1 diabetes mellitus
