# Supplementary material for: The Validation of the Parental Self-Efficacy Scale for Diabetes Management Among Parents of Children Wearing a Continuous Glucose Monitoring Sensor
Source: Biomedicines. 2025 May 27;13(6):1309. doi: 10.3390/biomedicines13061309 (PMC12189731; doi:10.3390/biomedicines13061309)
Supplement: Supplementary file 1 [file biomedicines-13-01309-s001.zip › biomedicines-3642875-supplementary.pdf]

Table S1. Inter-item correlations (Spearman's Rho)

	**Item 1**	**Item 2**	**Item 3**	**Item 4**	**Item 5**	**Item 6**	**Item 7**	**Item 8**	**Total score**
Item 1	1								0.7325
Item 2	0.6021	1							0.7409
Item 3	0.3823	0.3946	1						0.6782
Item 4	0.3744	0.4898	0.6225	1					0.6921
Item 5	0.4046	0.4437	0.5987	0.5817	1				0.7434
Item 6	0.4674	0.5900	0.3924	0.4352	0.4312	1			0.6941
Item 7	0.5809	0.5536	0.3745	0.4567	0.4667	0.5234	1		0.7908
Item 8	0.4822	0.4295	0.4825	0.4372	0.5372	0.3590	0.5563	1	0.7340

All correlations are significant at the $p<0.05$ level

Table S2. The item-level distribution of standard questionnaires and HbA1c values

	N	%	Chew	EQ-5D-5L	ICECAP-A	PedsQL Diab	HbA1c
Total	106	100.0					
Item 1[a]			p=0.009	p=0.021	p=0.002	p=0.368	p=0.029
Strongly Agree	1	0.9	1.0 (0.0)	1.0 (0.0)	0.8 (0.0)	85.7 (0.0)	7.1 (0.0)
2	8	7.5	4.0 (1.7)	0.9 (0.1)	0.8 (0.2)	72.3 (12.0)	7.6 (1.0)
3	15	14.2	3.2 (1.9)	0.9 (0.1)	0.9 (0.1)	69.3 (13.6)	7.7 (1.0)
4	21	19.8	1.9 (1.3)	1.0 (0.1)	0.9 (0.2)	75.5 (9.6)	7.6 (1.1)
Strongly Disagree	61	57.5	2.0 (1.7)	1.0 (0.0)	0.9 (0.1)	75.9 (11.4)	7.0 (0.9)
Item 2[a]			p=0.028	p=0.024	p=0.000	p=0.208	p=0.003
Strongly Agree	2	1.9	5.0 (2.8)	0.9 (0.0)	0.7 (0.3)	64.7 (0.6)	8.4 (0.1)
2	8	7.5	3.6 (1.7)	1.0 (0.0)	0.8 (0.1)	70.5 (13.3)	7.4 (1.0)
3	15	14.2	2.7 (1.4)	0.9 (0.1)	0.9 (0.1)	70.8 (12.8)	7.9 (0.9)
4	35	33.0	1.9 (1.5)	1.0 (0.1)	0.9 (0.1)	74.4 (12.3)	7.4 (1.0)
Strongly Disagree	46	43.4	2.1 (1.9)	1.0 (0.1)	0.9 (0.1)	77.4 (9.9)	6.9 (0.8)
Item 3			p=0.162	p=0.041	p=0.000	p=0.072	p=0.000
Strongly Disagree	0	0.0	0.0 (0.0)	0.0 (0.0)	0.0 (0.0)	0.0 (0.0)	0.0 (0.0)
2	1	0.9	1.0 (0.0)	1.0 (0.0)	0.8 (0.0)	70.5 (0.0)	8.5 (0.0)
3	9	8.5	2.9 (1.2)	1.0 (0.0)	0.8 (0.1)	71.7 (12.3)	8.4 (1.1)
4	53	50.0	2.5 (1.9)	0.9 (0.1)	0.9 (0.1)	72.3 (12.1)	7.5 (0.9)
Strongly Agree	43	40.6	1.9 (1.6)	1.0 (0.0)	0.9 (0.0)	78.4 (10.0)	6.8 (0.7)
Item 4			p=0.006	p=0.170	p=0.022	p=0.249	p=0.006
Strongly Disagree	1	0.9	2.0 (0.0)	1.0 (0.0)	0.7 (0.0)	75.0 (0.0)	7.8 (0.0)
2	1	0.9	1.0 (0.0)	1.0 (0.0)	0.8 (0.0)	70.5 (0.0)	8.5 (0.0)
3	8	7.5	3.4 (1.3)	1.0 (0.0)	0.9 (0.1)	65.6 (13.1)	7.7 (0.9)
4	33	31.1	3.0 (1.8)	0.9 (0.1)	0.9 (0.1)	74.0 (11.8)	7.7 (1.1)
Strongly Agree	63	59.4	1.8 (1.6)	1.0 (0.1)	0.9 (0.1)	76.3 (11.0)	7.0 (0.8)
Item 5			p=0.035	p=0.593	p=0.000	p=0.157	p=0.000
Strongly Disagree	0	0.0	0.0 (0.0)	0.0 (0.0)	0.0 (0.0)	0.0 (0.0)	0.0 (0.0)
2	2	1.9	1.5 (0.7)	1.0 (0.0)	0.8 (0.1)	72.8 (3.2)	8.2 (0.5)
3	12	11.3	3.3 (1.8)	1.0 (0.1)	0.8 (0.1)	72.6 (14.9)	8.0 (0.5)
4	56	52.8	2.4 (1.6)	1.0 (0.1)	0.9 (0.1)	72.7 (11.5)	7.4 (1.0)
Strongly Agree	36	34.0	1.8 (1.9)	1.0 (0.0)	1.0 (0.0)	78.7 (9.9)	6.9 (0.8)
Item 6[a]			p=0.128	p=0.082	p=0.000	p=0.068	p=0.063
Strongly Agree	2	1.9	3.5 (4.9)	0.9 (0.1)	1.0 (0.1)	69.2 (5.7)	7.7 (0.8)
2	1	0.9	4.0 (0.0)	0.9 (0.0)	0.8 (0.0)	86.6 (0.0)	7.4 (0.0)
3	13	12.3	2.8 (1.5)	0.9 (0.1)	0.8 (0.1)	66.6 (12.5)	7.7 (0.6)
4	28	26.4	2.6 (1.8)	1.0 (0.1)	0.8 (0.1)	77.5 (9.6)	7.5 (1.1)
Strongly Disagree	62	58.5	1.9 (1.6)	1.0 (0.1)	0.9 (0.1)	75.2 (11.6)	7.1 (1.0)
Item 7[a]			p=0.064	p=0.102	p=0.015	p=0.417	p=0.000
Strongly Agree	2	1.9	6.0 (1.4)	0.9 (0.1)	0.9 (0.0)	62.1 (4.4)	7.8 (0.7)
2	16	15.1	2.7 (1.7)	0.9 (0.1)	0.8 (0.1)	75.4 (11.7)	7.9 (1.1)
3	14	13.2	2.6 (1.3)	1.0 (0.1)	0.8 (0.1)	70.8 (14.5)	7.8 (0.8)
4	22	20.8	2.1 (1.7)	1.0 (0.1)	0.9 (0.2)	74.3 (11.4)	7.4 (0.9)
Strongly Disagree	52	49.1	2.0 (1.8)	1.0 (0.1)	0.9 (0.0)	76.2 (10.7)	6.9 (0.8)

	N	%	Chew	EQ-5D-5L	ICECAP-A	PedsQL Diab	HbA1c
Item 8			p=0.279	p=0.140	p=0.009	p=0.242	p=0.002
Strongly Disagree	1	0.9	4.0 (0.0)	1.0 (0.0)	0.9 (0.0)	86.6 (0.0)	6.8 (0.0)
2	1	0.9	3.0 (0.0)	1.0 (0.0)	0.7 (0.0)	79.5 (0.0)	8.6 (0.0)
3	16	15.1	2.8 (1.4)	0.9 (0.1)	0.8 (0.2)	68.9 (12.8)	8.0 (0.8)
4	56	52.8	2.3 (1.7)	1.0 (0.1)	0.9 (0.1)	75.7 (10.9)	7.3 (1.1)
Strongly Agree	32	30.2	2.0 (2.0)	1.0 (0.0)	0.9 (0.0)	75.4 (11.6)	6.9 (0.7)

[a] Items are reversely scored

Differences by subgroups were tested with the Kruskal-Wallis test

The eight items of the PSESDM by Marchante et al. 2014:

1. It is hard for me to find ways to solve problems that occur in dealing with my child’s diabetes.
2. When I try to change things I don’t like about my child’s diabetes, it doesn’t work.
3. I take care of my child well when it comes to his/her diabetes.
4. I am able to deal with things related to my child’s diabetes as well as others.
5. I am successful when it comes to projects I do to take care of my child’s diabetes.
6. Usually, my plans to take care of my child’s diabetes don’t work out.
7. No matter how hard I try, taking care of my child’s diabetes doesn’t turn out the way I like.
8. I’m usually able to accomplish the goals I set in trying to take care of my child’s diabetes.
